# Dental Education

**Published:** 1891-06

**Authors:** Henry Leffmann


### DENTAL EDUCATION.[1]

BY HENRY LEFFMANN, M.D., D.D.S.[2]

For some years a wholesale agitation has been in progress concerning the necessity of improving the standard of education in those professions which have to do with the prevention or treatment of disease or with procedures affecting the rights and privileges of the citizen. The medical profession proper has, of course, received the greatest share, but some attention has been given to the question of advancement in dental education and to the importance of establishing exact methods of instruction in hygiene and medical jurisprudence. No doubt the day is not far distant when we will see special degrees granted in these branches, after definite courses of study and examination.

Dentistry has enjoyed among all the specialties of medicine what might be called an "evolutionary" advantage, it originated without the domain of medicine proper, and has been growing into it slowly, and as when some town or country, with special political privileges, annexing itself to a larger territory, carries with it by agreement certain rights, so the science of dentistry is vouchsafed a degree of independence much greater than is accorded to those specialties which are just budding from the parent stem. No one is disturbed at the conferring of the special degree of doctor of dental surgery; but the corresponding degrees of doctor of ophthalmology, otology, dermatology, are yet in abeyance, and the proposition to confer them meets either with opposition or indifference.

I have always regarded with satisfaction the fact that I was one of a small company of dentists that assembled in New York City a few years ago and started the movement for advance in the thoroughness on dental education by terminating the one year system, and establishing the compulsory two years' course. The results of that reform have been very gratifying; all who teach in dental colleges see the great advantage that accrues when the student is given ample time for acquiring a knowledge of the science and art of the profession which he is to practise. At the present time we are on the threshold of another extension of the

---

[1] Read before the Pennsylvania State Dental Society, July 29, 1890.

[2] Professor of Chemistry in the Pennsylvania College of Dental Surgery.

course, namely, to three years. While I have no doubt that such extension will be of benefit, yet I think it may be a question whether there might not have been general reforms brought about in other features in the course of instruction more urgent than is the further extension of the lecture course.

The principal obstacle to advancement in this field is the difficulty of securing uniformity, and an honest adherence to the proposed plans. The majority of the medical and dental colleges of the United States are unendowed and dependent on their students for support. However disinterested the utterances may be, it is not in accordance with general human nature that any faculty should deliberately cut down its revenues by wholesale unless under some strong public pressure or special emergency. The establishment, therefore, of preliminary examinations and higher standards of final examinations comes but slowly, for as the sole judgment on such matters rests with the individual college, there will be always abundant opportunity for uncrupulous men to deal unfairly with their co-workers, and bring about what has been well called "the downward competition of the schools." It is possibly for this reason that the standards fixed for the colleges have generally been such that evasion is difficult. A college may, for instance, advertise that it requires a preliminary examination and a high standard of final examination, but it will be difficult to make certain of these; but it can, however, be seen at once, if it has a certain number of branches represented in its curriculum, and requires a certain term of study and a certain number of such terms.

It is curious that, just as the medical colleges are agitating the advisability of increasing the course of study to four years, and the dental colleges about entering upon a three years' system, some of the leading colleges in arts and general science are advocating a shortening of the course for academic degrees from four to three years. The reason alleged is that many men can acquire in three years what others will only acquire in four, and that it is unjust to the better class to compel them to lose a year. The same argument applies to those who enter for professional degrees. Many can absorb rapidly all the theoretical instruction, and their progress is necessarily delayed by the weaker classmates. There is, however, in the academic institutions, generally, a condition which secures the rapid and successful progress of the student to an extent not yet available in colleges conferring professional degrees; that is, the extent and stringency of the preliminary examination. If a young man or woman desires to enter for one of the degrees in arts or sciences at a reputable college, a definite amount of preparation is necessary, and when once entered upon the list, the teaching faculty can make a close estimate of the capacity of every member of the class, and each member is aware of the degree to which he can presume a knowledge of the fundamental principles of the science he may be teaching. Thus, if the entrance examination include a knowledge of elementary physics, it will not be necessary to stop to explain the significance of specific gravity, nor the nature of a thermometer. Such aids are, with very few exceptions, not at hand in the colleges for conferring professional degrees. It is true that with many institutions an effort has been made towards preliminary requirements, and some good has been accomplished in this manner, but it has, I think, been operative largely in deterring unfit candidates rather than raising the general standard. We still find, side by side upon the same benches, the student who has read Cæsar and solved quadratic equations and the student who does not know what is meant by a genitive case and has no idea of abstract quantities.

The remedy for these deficiencies lies, I believe, in State supervision. I regret that I must come to this conclusion, for I have always been opposed to interference by the constituted authorities with the general conduct and freedom of the citizen. But no one can fail to note the beneficial effect which has been exercised by State Boards of Examiners, and it will be of advantage when their powers are further extended. We have waited many years for the colleges to come up to the same high standard, and it will be necessary to force them up to it. What is needed in the case is uniformity and definiteness in the requirements, and this can only come when those requirements are determined by legalized authorities and enforced without fear or favor on all who wish to enter the profession. The sole duty of the college is to teach; it should be relieved from the necessity of soliciting students or granting them license to practise. The latter function is a prerogative of the State, and should be reserved to it.

If we turn for a moment to the practical example of how colleges have to deal with the problem of increasing the term of study, we find that in most cases a serious mistake has been made. One of the reasons for the increase has been the necessity of more thorough instruction in the theoretical branches, and yet, in most cases, the amount of time given to such branches is not materially increased, nor are the requirements for entering upon their study advanced. If a college changes from two to three years, it will be found that, very probably, the professor of chemistry or physiology will not have any more lectures. The principal change will be that the student will drop these studies in the third year, and that the whole work will be spread over an extra year. It is true that there will be less crowding, and more opportunity for accessory reading, but, as a matter of fact, the most valuable teaching is that which is done in the laboratory and lecture-room, and the student's own studying is largely for examination. The various fundamental branches which make up a course in medical or dental science should be carried through the entire course. Some of the applications of physiology and chemistry are only to be appreciated when the more advanced details of the practical departments have been acquired. A proper arrangement of the extended course is, it seems to me, to use the extra year as a preparatory year. Instead of making a third (or fourth) year devoted only to the so-called practical branches, this year should, as heretofore, include all the fundamental branches, but the first year should be exclusively devoted to preparation for the study. Thus, in a dental college, I would not have any clinical work done during the first year, but the principles of physiology, histology, anatomy, chemistry, and metallurgy should be taught. Practical anatomy should be limited either to the study of the manikin, or the dissection of some of the lower animals; both of these methods are more economical and satisfactory with new students than the use of human cadavers. In the second year the application of chemistry to physiology and pathology should be begun, materia medica, prosthetic dentistry, dissection of the human body, dental physiology, pathology, and dental practice. The third year should be devoted to operative dentistry and oral surgery, therapeutics, and organic chemistry, with special lectures on dental hygiene, etiology, and prophylaxis. At the end of each year examinations should be held to determine proficiency in each branch, and students should be compelled to acquire a reasonable degree of knowledge before being passed in any branch, and under no circumstances should proficiency in one branch be permitted to atone for complete deficiency in another. I also think very well of the plan of setting before the more able pupils some inducement to greater work than the curriculum calls for, by conferring the degree *cum laude* upon such as comply with extra requirement.

So far as the preliminary examination is concerned, it should include a knowledge of such branches as will aid the student in understanding the lectures. From an experience in this field, extending over twenty-one years, I am satisfied that a great many pupils understand much less of the subjects they study than even is commonly supposed. I have found, especially, a deficiency in elementary arithmetic, which is necessary to the comprehension of many demonstrations in chemistry and metallurgy. Spelling and grammar are, of course, always defective, and the preliminary examination should include some work in those topics. Unless the college course provides for a definite course in physics during the first year, some knowledge of the elementary physics of light, heat, and electricity, and specific gravity should be insisted upon. As a rule, it will be found that the only way in which to secure uniformity in such standards of admission is to test them by actual examination, which should be in writing, public, and of record. Certificates of proficiency, unless of very definite character, are apt to be misleading.

So far as the final examinations are concerned, I hold that in dental as in medical colleges they are matters for the State. The duty of the faculty ceases when the pupil has been given a conspectus of the present state of the art and science of his chosen profession. It is highly advisable, of course, that the college should test by examination the degree of proficiency by any method it may see fit, and that it should be empowered to give any form of certificate to attest this fact, but the entry upon the practice of the profession is an act which concerns the community, and to it, therefore, belongs the exclusive right of permission. Every guard should, of course, be thrown around the administration of this right. It should be public, uniform, and of record, and should be under the partial supervision at least of the teaching bodies, but not controlled by them.

Unfortunately, for the reasons that I have pointed out, many of these reforms are not immediately attainable. Much opposition yet exists to official control, and some colleges continue to profess a desire to enforce a high standard; but the knowledge that these professions cannot be sincere prevents other institutions from discarding old methods. We must await a gradual development of public opinion, but I am confident that a good preliminary examination, a graded course arranged in accordance with the requirements of modern science, and a State board of examiners with sole power to admit to practice will accomplish all that is needed in dental education.

